# Study of Elastic and Structural Properties of BaFe_2_As_2_ Ultrathin Film Using Picosecond Ultrasonics

**DOI:** 10.3390/ma16217031

**Published:** 2023-11-03

**Authors:** Di Cheng, Boqun Song, Jong-Hoon Kang, Chris Sundahl, Anthony L. Edgeton, Liang Luo, Joong-Mok Park, Yesusa G. Collantes, Eric E. Hellstrom, Martin Mootz, Ilias E. Perakis, Chang-Beom Eom, Jigang Wang

**Affiliations:** 1 Department of Physics and Astronomy, Iowa State University, Ames, IA 50011, USA; dcheng@iastate.edu (D.C.); bqsong@iastate.edu (B.S.); liangluo@ameslab.gov (L.L.); joongmok@iastate.edu (J.-M.P.); 2 Ames National Laboratory-USDOE, Ames, IA 50011, USA; 3 Department of Materials Science and Engineering, University of Wisconsin-Madison, Madison, WI 53706, USA; jkang@postech.ac.kr (J.-H.K.); sundahlc@gmail.com (C.S.); edgeton@wisc.edu (A.L.E.); ceom@wisc.edu (C.-B.E.); 4 Applied Superconductivity Center, National High Magnetic Field Laboratory, Florida State University, Tallahassee, FL 32310, USAhellstrom@asc.magnet.fsu.edu (E.E.H.); 5 Department of Physics, University of Alabama at Birmingham, Birmingham, AL 35294-1170, USA; mootz@iastate.edu (M.M.); iperakis@uab.edu (I.E.P.)

**Keywords:** ultrathin film, BaFe_2_As_2_, intrinsic strain, longitudinal acoustic phonon, through-thickness elastic stiffness coefficient (*C*_33_)

## Abstract

We obtain the through-thickness elastic stiffness coefficient (*C*_33_) in nominal 9 nm and 60 nm BaFe_2_As_2_ (Ba-122) thin films by using picosecond ultrasonics. Particularly, we reveal the increase in elastic stiffness as film thickness decreases from bulk value down to 9 nm, which we attribute to the increase in intrinsic strain near the film-substrate interface. Our density functional theory (DFT) calculations reproduce the observed acoustic oscillation frequencies well. In addition, temperature dependence of longitudinal acoustic (LA) phonon mode frequency for 9 nm Ba-122 thin film is reported. The frequency change is attributed to the change in Ba-122 orthorhombicity (a−b)/(a+b). This conclusion can be corroborated by our previous ultrafast ellipticity measurements in 9 nm Ba-122 thin film, which exhibit strong temperature dependence and indicate the structural phase transition temperature *T*_s_.

## 1. Introduction

The elastic properties of thin films are of vital importance as they play key roles in determining the performance and long-term stability of thin film devices such as thin film superconductors, halide perovskites, and spintronics devices [1,2,3,4,5,6]. As film thickness decreases, the lattice-substrate mismatch effect and interface strain are enhanced. As a result, the electronic and elastic properties of thin films can significantly deviate from their bulk counterparts.

Recent studies have shown that the critical temperature (*T*_c_) of thin film superconductors is susceptible to modulation through variations in film thickness and substrate composition [7,8,9,10,11]. In this realm, the undoped Fe pnictide BaFe_2_As_2_ (Ba-122) thin film emerges as an ideal platform for studying the thickness-dependent superconductivity. Bulk Ba-122 is non-superconducting and its superconductivity can be generated and greatly enhanced by lowering the film thickness down to sub-10 nm [10,11]. For these thin film superconductors, elastic properties are important as they not only influence the device functionality but also pave the way for extracting other key information such as structural phase transition and electron–lattice coupling. Despite the extensive efforts devoted to investigating the electronic and superconducting properties of thin film superconductors [1,7,9,12], pertinent studies on elastic properties are rare, which has motivated the present study on Ba-122 thin films.

To extract elastic properties of sub-10 nm thin films, our methodology harnesses picosecond ultrasonics, specifically the generation and detection of high-frequency coherent acoustic phonons via ultrashort laser pulses. After the pioneering work in 1984 by Thomsen et al. [13], enduring efforts have been dedicated to generating and studying phonon modes using ultrafast lasers [14,15,16,17,18,19,20,21,22,23,24]. In this methodology, an intense ultrafast optical pulse (pump) disturbs the thin film, resulting in the generation of a strain pulse. This pulse then propagates through the film, eventually encountering the film-substrate interface. Due to the acoustic mismatch between the thin film and the substrate, a fraction of this strain pulse reflects, while the remainder either gets absorbed at the interface or continues its propagation through the substrate. Both the reflected and transmitted strain pulse may cause a periodic modulation of optical properties as will be subsequently measured by a time-delayed light pulse (probe) at the sample’s surface. The detected modes are usually LA waves in contrast to shear waves, i.e., transverse acoustic (TA) waves. The generation of the latter is restricted to thermally anisotropic materials and arises from anisotropic thermal expansion [25]. Picosecond ultrasonics is adept at examining thin films and layered configurations. Compared to conventional methods such as nanoindentation and atomic force microscopy whose thresholds are limited by probe radius and indentation depth [26,27,28,29], picosecond ultrasonics offers a non-destructive approach combined with unprecedented sensitivity, time resolution, and applicability in ultrathin films thinner than 10 nm.

The Ba-122 phase of iron pnictide materials hosts a series of layered intermetallics [30,31,32] and their derivatives [33,34], subject to a tetragonal-orthorhombic transition and a phase diagram involving magnetism and superconductivity [35]. The increase in Ba-122 thin film’s interface strain, which manifests itself in elastic properties change, gives rise to the decrease in its orthorhombicity. The orthorhombicity change can then be referred from the picosecond ultrasonics measurement; it can also be corroborated by ellipticity measurement, which measures the complex rotation angle of a linearly polarized light reflected off a material with two-fold in-plane anisotropy, the quantity that changes with sample’s orthorhombicity. A more detailed description of the ellipticity setup and measurement methods can be found in ref. [31].

In this letter, we measure the elastic stiffness coefficient (*C*_33_) and sound speed of Ba-122 thin films using picosecond ultrasonics. This is implemented by ultrafast two-color transient reflectivity measurement utilizing a femtosecond 800 nm (1.55 eV) pump and 400 nm (3.10 eV) probe laser pulses. Nominal 9 nm and 60 nm Ba-122 thin films were measured and compared (For convenience, the term “nominal” will be omitted in the following discussion).

## 2. Materials and Methods

Epitaxial Ba-122 thin films were grown on single-crystal substrate (001) lithium fluoride (LiF) by pulsed laser deposition using a custom-made deposition system with a 248 nm KrF ultraviolet excimer laser (Coherent LPXpro 305, Santa Clara, CA, USA) at 650–700 ${}^{{}^{\circ}}$C. Throughout the growth procedure, the repetition rate of the KrF laser was consistently maintained at 40 Hz.

**Figure 1:**
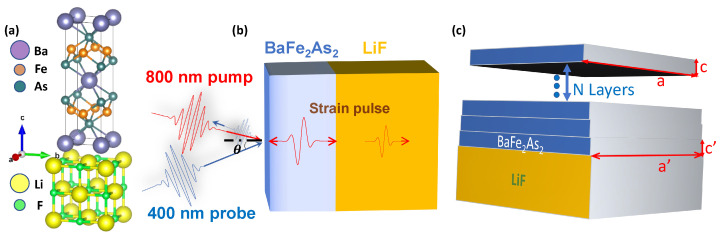
Scheme of ultrafast pump-probe measurement and information of sample: (**a**) Crystal structures of Ba-122 and LiF at room temperature [40]. (**b**) Generation and detection of coherent acoustic phonons in Ba-122 thin films. The coherent phonons generated by the 800 nm pump pulse can be detected by the time-delayed 400 nm probe pulse through the photoelastic effect. (**c**) Schematics of *a*–*c* over layers for Ba-122 thin film. Note the $a^{\prime }$ and $c^{\prime }$ denote the lattice parameters near the LiF substrate.

In the picosecond ultrasonics measurement, the output from a Ti:Sapphire amplifier (Spectra-Physics Spitfire, Santa Clara, CA, USA) with 800 nm center wavelength, 40 fs pulse duration, and 1 kHz repetition rate was split into two beams. As shown in Figure 1b, the first pump beam, used for photoexcitation, was focused onto the sample with a 150 μm diameter and 0.7 μJ pulse energy, leading to a pump fluence of approximately 4 mJ/cm^2^. The second probe beam, frequency-doubled to 400 nm via a beta Barium Borate (β-BBO) crystal, was focused onto the sample with a diameter of 50 μm. The pump-induced probe reflectivity change was measured as a function of pump-probe delay time, which was controlled by an optical delay stage. Both the pump and probe beams were incident on the thin films at a nearly normal incidence.

Figure 1a displays the crystal structures of both Ba-122 and LiF. Figure 1b illustrates the technique used to generate LA phonons and detect them on the Ba-122 thin film’s surface. Figure 1c depicts the arrangement of *a*–*c* over layers in the Ba-122 thin film. At room temperature, the lattice constants for the Ba-122 single crystal are *a* = *b* = 0.3961 nm and *c* = 1.3020 nm. These values correspond to a tetragonal structure, which is indexed under the *I4/mmm* space group [36], and upon cooling, the structure undergoes a phase transition to an orthorhombic phase, characterized by the space group *Fmmm* [37]. In contrast, LiF adopts a face-centered cubic structure with the space group $Fm\bar{3}m$ and has lattice parameters *a* = *b* = *c* = 0.40173 nm at room temperature [38]. Notably, LiF does not undergo any phase transition upon cooling [39]. In the proximity of the LiF substrate, the lattice constants of the Ba-122 thin film adjust significantly, aligning more with those of LiF. This shift can be attributed to the lattice-substrate mismatch effect and interface strain. However, the upper layers of Ba-122 films are much less influenced by the substrate, retaining lattice constants that align closely with the bulk material’s values.

## 3. Results and Discussion

### 3.1. Low Temperature Transient Reflectivity Change

**Figure 2:**
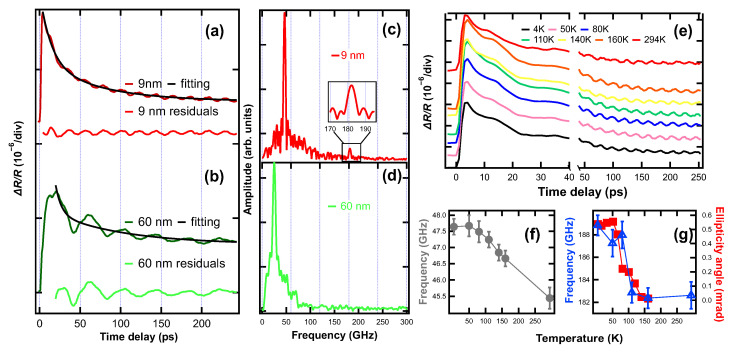
Transient reflectivity change ΔR/R with corresponding biexponential fitting and residuals of (**a**) 9 nm and (**b**) 60 nm Ba-122 thin film at 160 K. Data are offset for clarity. Fourier spectra for the residuals of (**c**) 9 nm and (**d**) 60 nm Ba-122 thin film transient reflectivity, insets show the zoom-in data with frequency ranges from 170 to 195 GHz. Clearly, the peak at 182.6 GHz in (**c**) is absent in (**d**). (**e**) 9 nm Ba-122 thin film temperature-dependent transient reflectivity ΔR/R change (with offset) and acoustic phonon frequency change for the (**f**) first dominant peak and (**g**) second peak (blue triangles) plotted together with temperature-dependent photoinduced ellipticity amplitude (red squares) [11]. The transition temperature has a range of ∼110–160 K.

Figure 2a,b show the transient reflectivity ΔR/R of 9 and 60 nm Ba-122 thin films at *T* = 160 K, respectively. Besides the exponential decay of the ΔR/R signal, periodic oscillations in ΔR/R were observed in both samples. The acoustic pulse generated at the surface propagates through the oscillations of the thin film’s sensitivity function, which gives rise to the observed oscillations. Through the fitting analysis of the ΔR/R data, we observed that a single exponential decay model fails to adequately match the experimental results. However, a biexponential decay model works pretty well. The decay can be described as $\Delta R/R=R_{0}+A_{1}\exp^{-(t-t_{0})/\tau_{1}}+A_{2}\exp^{-(t-t_{0})/\tau_{2}}$, where $t_{0}$ is the starting time for exponential decay, $R_{0}$ is a time-independent constant, $A_{1}$ and $A_{2}$ are the amplitudes of each exponential term, *t* is the time delay, and $\tau_{1}$ and $\tau_{2}$ are the decay time for the first and second exponential terms, respectively. The fitted $\tau_{1}$ and $\tau_{2}$ are 14 ps (7 ps) and 78 ps (177 ps) for 9 nm (60 nm) thin film. Following the subtraction of the biexponential decay background, we conducted a fast Fourier transform (FFT) analysis on the residual transient reflectivity. There are two peaks for 9 nm thin film. The first dominant peak is located at ∼46.6 GHz and the second peak is observed at ∼182.6 GHz (Figure 2c). In the FFT spectrum of the 60 nm thin film, only a singular peak at ∼25.0 GHz is observed (Figure 2d), and the oscillation undergoes gradual damping (Figure 2b). The signal generated within the LiF substrate should be long-lived as LiF is almost transparent for a 400 nm probe. However, the signals generated within Ba-122 thin films should be short-lived as part of the strain pulse transmits into the substrate each time it reaches the film-substrate interface. Through comparison with the documented longitudinal sound velocity of LiF (which will be presented subsequently), the 46.6 GHz peak in 9 nm thin film is attributed to the LA phonon transmitted into the LiF substrate. Accordingly, the 182.6 GHz (25.0 GHz) peak is ascribed to the generation of the LA phonon within the 9 nm (60 nm) thin film. The rationale behind the absence of the 46.6 GHz peak in the 60 nm thin film will be elucidated in a subsequent discussion.

The signals are generated through two mechanisms. For the signal generated within a sufficiently thick LiF substrate, the detected oscillation is due to the interference between the light reflected from both sides (mainly the front surface) of thin films and the light reflected from the strain pulse propagating through the substrate along one direction (Brillouin scattering). The acoustic phonon frequency $f_{a}$ can be determined by the constructive interference conditions: $2n\cdot \left(u/f_{a}\right)\cdot \cos\left(\theta \right)=g\cdot \lambda_{p}$, where *n* is the refractive index of the substrate, *u* is the sound speed, θ is the incidence angle of the probe beam (in our experiment the θ is close to zero), $\lambda_{p}$ is the probe wavelength 400 nm, and g=0,1,2…. Thus, for the first order g=1, the resonance phonon frequency is expressed $f_{a}=2n\cdot u\cdot \cos\left(\theta \right)/\lambda_{p}$ [18]. The refractive index *n* for LiF at 400 nm (25,000 cm^−1^) is 1.4 [41]. We can calculate the sound velocity of the substrate u=6657 m/s, which is in good agreement with the reported value (6750 m/s at 160 K) [42].

**Figure 3:**
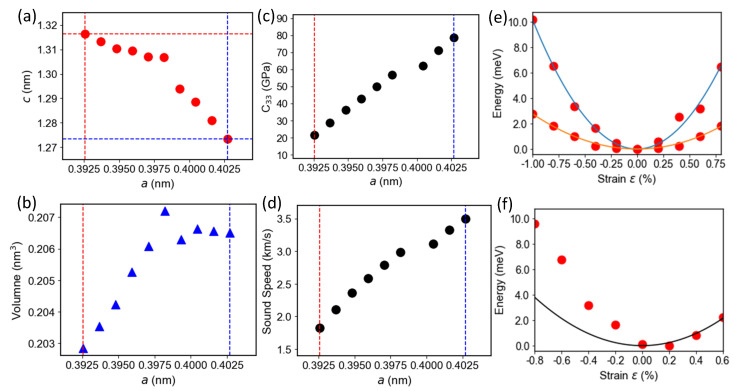
Calculated DFT results with different parameters: (**a**) Lattice parameter *c* as a function of lattice parameter *a*. (**b**) Cell volumes as a function of lattice parameter *a*. (**c**) *C*_33_ dependence of *a*. (**d**) Sound speeds as a function of lattice parameter *a*. (**e**) Strain energy function *E* for 9 nm thin film (red dots, solid blue line is fitting) and bulk (red dots, solid red line is fitting). (**f**) Energy as a function of strain ϵ when *a* = 0.399 nm (red dots, solid black line is fitting).

For the signals generated within the Ba-122 thin film, the oscillations are caused by the interference of light reflected from the front surface and from the strain pulse which bounces back and forth within the thin film, i.e., local resonances. Hence, one period of the oscillation corresponds to a round trip time of the strain pulse, and the sound speed is given by $f_{a}=mu/2d$ when the acoustic impedance of thin film is larger than that of the substrate, where *d* is the thin film thickness, *m* is the mode index number and *u* is the sound speed. Otherwise, if the substrate has a greater impedance, $f_{a}=\left(2m-1\right)u/4d$. Because we could only see the first order and, as will be seen in a later discussion, the thin films indeed have greater acoustic impedance, we use $f_{a}=u/2d$. To accurately determine the calculated sound speed, we used X-ray reflectivity (XRR) and transmission electron microscopy (TEM) to measure the thickness of the two films, respectively, which yielded d=9.1 ± 0.1 nm and d= 62.4 ± 1.2 nm (see Supplementary Materials for detailed thickness measurements). With $f_{a}=$182.6 GHz ($f_{a}=25.0$ GHz) and precisely determined film thicknesses, we find u=3323 ± 37 m/s (u=3120 ± 60 m/s) for 9 nm (60 nm) thin film. Note that the skin depth of Ba-122 at 400 nm is given by: $\delta =\sqrt{1/\pi f\mu \sigma }$ [43], where *f* is the frequency of probe beam (400 nm), μ is the permeability of Ba-122 (∼$\mu_{0}$, which is $4\pi \times 10^{-7}$ N/A2), and σ is the optical conductivity of Ba-122 at 400 nm. With σ= 233,440 Ω^−1^/m^−1^ [44] we find δ= 38 nm, which elucidates the reason for the absence of the 46.6 GHz peak in the 60 nm thin film. The sound velocity is related to through-thickness coefficient $C_{33}$ through the equation $u=\sqrt{C_{33}/\rho }$, with ρ being the sample density. By comparing the obtained sound velocities with Figure 3d, the overall lattice parameter *a* for 9 nm (60 nm) thin films is ∼0.4015 (0.3980) nm and the corresponding unit cell volume is 0.2065 (0.2062) nm^3^ (Figure 3b). Thus, two thin films differ by ∼0.14% in their densities, which is insignificant compared to the difference in their sound velocities (∼6.5%) and the main difference in calculated sound velocities is from the difference in $C_{33}$. Considering the density values ρ = 6.48 (2.64) g/cm^3^ for Ba-122 (LiF), the corresponding acoustic impedance Z = ρ*u* for the 9 nm (60 nm) Ba-122 thin film is calculated as 21.53 (19.59) MPa· s/m, which is higher than the acoustic impedance of LiF (17.57 MPa· s/m). Here, Z represents the acoustic impedance and *u* denotes the sound speed. The acoustic amplitude reflection coefficient Γ at the interface between the thin film and substrate is determined by the formula: Γ = (Z_film_−Z_sub_)/(Z_sub_ + Z_film_). For the 60 nm Ba-122 thin film, Γ is ∼0.05. This indicates that only around 5% of the strain pulse will be reflected from the interface during each occurrence, resulting in an overdamped oscillation. However, in the experimental observations, the oscillations within the 60 nm thin film only display slight damping, characterized by a gradual decrease in amplitude. One potential explanation for this discrepancy is that the sound speed values obtained represent average values for the entire thin film. However, at the interface, the lattice-substrate effect and interface strain are more pronounced compared to other regions, resulting in an elevated acoustic impedance within the Ba-122 thin film and a larger reflection coefficient. Additionally, the slow damping characteristics indicate a high-quality surface and interlayer condition, as supported by the TEM measurements (see Supplementary Materials). A poor interface or surface roughness would usually shorten the oscillation duration. Moreover, the narrow bandwidth of the 183 GHz peak (∼4.9 GHz) suggests strong coherence in the LA generated within the film, showing the film’s uniformity and minimal surface/interface roughness.

### 3.2. Temperature Dependent Transient Reflectivity Change

In order to investigate the structural phase transition of the 9 nm Ba-122 thin film, we conducted temperature-dependent measurements of the LA phonon frequency. Prior studies [45,46] have already examined this behavior across the phase transition in Ba-122 single crystals. Figure 2e shows the transient reflectivity ΔR/R of the 9 nm film at temperatures ranging from 4 to 294 K. The temperature-dependent LA phonon mode frequency for the first and second peaks extracted from Figure 2e are shown in Figure 2f,g. Figure 2f illustrates that the frequency of the predominant LA phonon generated within the LiF substrate exhibits a linear increase from 294 to 50 K, followed by a nearly constant behavior from 50 to 4 K. This observed trend aligns well with the temperature-dependent behavior of $C_{33}$ in LiF [42]. In Figure 2g, it can be observed that the LA phonon mode frequency and the photoinduced ellipticity amplitude of the 9 nm thin film exhibit a state of relative stability from 294 K to 160 K, followed by an increase commencing at ∼160 K. Notably, a significant and rapid change is observed in the temperature range of 110 to 80 K, with subsequent cooling resulting in a diminishing rate of change. Based on Figure 2g, *T*_s_ should be in between 110 K and 160 K. Note that 136 K [45] and 134 K [46]*T*_s_ are reported for bulk Ba-122. The photoinduced ellipticity amplitude is directly proportional to the two-fold in-plane anisotropy in Ba-122 thin film and the change of ellipticity amplitude is consistent with the change of sample’s orthorhombicity as reported in detail in our prior study [11], which means that the change of LA phonon mode frequency is well correlated to the change of the orthorhombicity of Ba-122 thin film. Therefore, we ascribe the reduction in the frequency of the LA mode generated within the Ba-122 thin film to the corresponding decrease in $C_{33}$, which can be traced back to the diminishing orthorhombicity of the material.

## 4. Theory and Calculation

To understand *u* at different film thicknesses, we performed density functional theory (DFT) calculations [47] with a set of tested parameters. Note that the sound propagates perpendicular to the film surface and thus mainly depends on the elastic properties along the *c*-axis. On the other hand, the strain caused by the mismatch of substrate LiF (a=0.40173 nm) and Ba-122 (a=0.3961 nm) mainly affects the *a*–*b* plane, i.e., the tetragonal unit cell tends to be more expanded as it gets closer to the substrate, which is subject to intense distortion. The 9 nm film corresponds to 6 layers of the unit cell (Figure 1b); while the 60 nm film corresponds to 40 layers, which is close to a bulk state. With this insight, we model the influence of film thickness by the extension of distortion in the *a*-*b* plane (with tetragonal symmetry). In other words, we try to address the drastic change in *u* by an elastic effect. Our purpose is to examine whether the elastic distortion can possibly give rise to the significant changes observed in experiments.

We examine *a* in a range from 0.3925 nm (note, for bulk Ba-122, a is 0.3961 nm) to 0.40173 nm (the lattice parameter of LiF substrate). Figure 3a illustrates the lattice parameter *c* decreases with parameter *a*. Meanwhile, the cell volume *V* is not constant but will increase with *a* (Figure 3b). That suggests the bottom layer generally has a larger unit cell than the top (bulk). Elastic property, like *C*_33_, can be examined, which is expressed as [48]

(1) $$C_{33}=\frac{1}{V}\frac{\partial^{2}E}{\partial \varepsilon^{2}}.$$

*V* is the unit cell volume of the given distorted lattice *a*; *E* is the total strain energy, ε is the strain, which especially refers to $\varepsilon_{cc}$ in this scenario (the diagonal term along *c*-axis). With DFT and unit cell method, we can evaluate $\partial^{2}E/\partial \varepsilon^{2}$ by a fixed *a* (maintain symmetry) and a slightly varied *c* around the equilibrium $c_{eq}$ at given *a*.

(2) $$\varepsilon_{cc}=\Delta c/d,\;\Delta c=c-c_{eq}\left(a\right)$$

where *d* is the thickness of the thin film. The results are shown in Figure 3c,d. Notably, with increased *a* and *V*, $C_{33}$ undergoes a four-time-increase (Figure 3c). Combined with

(3) $$u=\sqrt{C_{33}/\rho }=\sqrt{C_{33}V/m},$$

we find that for bulk sample (*a* = 0.3961 nm), the sound speed is around 2400 m/s and when the strain is imposed, the sound speed keeps increasing to >3000 m/s (Figure 3d). DFT presents a correct trend for sound speeds at varied lattice distortions: sound speeds increase with strains, consistent with our observations. On the other hand, the lower end of theoretical values is noticeably lower than measured speeds, which could either indicate that both films significantly deviate from bulk situations or that the discrepancy is partially due to DFT’s underestimates. Which is the case required for further investigations.

Note that ε is evaluated for the whole film, rather than for different locations within a film. Thus, the evaluated $C_{33}$ physically means the average value for the whole thin film. It is important to realize one fitting curve (parabolic) in Figure 3e will only correspond to one point in Figure 3c. For example, in Figure 3e, the red fitting curve corresponds to the bulk limit (red dashed line in Figure 3c), and the points represent a series of small deviations around bulk; while the blue curve corresponds to the highly distorted limit (blue dashed lines in Figure 3c).

Noteworthy, discontinuity is observed when the lattice parameters are varied (Figure 3a–c,e). This discontinuity corresponds to a phase transition, which is relevant to the double (or multiple) energy valleys [49], which have been observed in layer-stacking structures, e.g., Ba-122/Fe-based 1144-phase [30]. It occurs when the *c* parameter decreases, either for an applied pressure or for an extended *a*–*b* plane as performed in this case. The *c* lattice parameter will undergo a drastic change at a critical value, and it is called the collapse phase [30]. Consequently, from Figure 3f, the strain energy function near the discontinuity is obviously deviant from the parabolic line. That means *C*_33_ will exhibit quite different values on the two sides. It is interesting to check such abnormality using nano-scales imaging [50,51] and other THz spectroscopy techniques [52,53,54,55].

## 5. Conclusions

To summarize, we discovered propagating coherent LA phonon wavepackets in Ba-122 thin films using picosecond ultrasonics. We revealed a clear difference in the excited LA mode frequency between 9 nm and 60 nm Ba-122 thin films. The stiffness coefficient $C_{33}$ of the 9 nm thin film is notably higher than its 60 nm counterpart. Temperature-dependent LA mode frequency and temperature-dependent ellipticity of 9 nm thin film correlate with the change of the sample’s orthorhombicity. We attribute the change in mode frequency to the change in orthorhombicity, which leads to the change in $C_{33}$, consistent with our calculations. Our results show that picosecond ultrasonics represents a powerful and sensitive tool for studying the elastic and structural properties of Ba-122 thin films. Furthermore, given known sound speeds, the technique facilitates non-destructive thickness measurements for ultrathin films. Such an approach can be extended to investigate various elastic and structural properties, including Young’s modulus, Poisson’s ratio, thermal diffusivity [56], and structural phase transitions, in a plethora of intricate thin films, encompassing superconductors [57,58], magnetics [59,60], and photovoltaics [61]. Moreover, the method’s acute sensitivity to elasticity changes allows for the detection of embedded features and anomalies in thin films [62,63].

## Data Availability

The data presented in this study are available on request from the corresponding author.

## Supplementary Materials

The following supporting information can be downloaded at: https://www.mdpi.com/article/10.3390/ma16217031/s1. Figure S1: Thickness determinations of Ba-122 thin films. Reference [64] is cited in supplementary materials.

## References

[1] Haindl S., Kidszun M., Oswald S., Hess C., Büchner B., Kölling S., Wilde L., Thersleff T., Yurchenko V., Jourdan M. (2014). Thin film growth of Fe-based superconductors: From fundamental properties to functional devices. A comparative review. Rep. Prog. Phys..

[2] Li Y., Polakovic T., Wang Y.L., Xu J., Lendinez S., Zhang Z., Ding J., Khaire T., Saglam H., Divan R. (2019). Strong coupling between magnons and microwave photons in on-chip ferromagnet-superconductor thin-film devices. Phys. Rev. Lett..

[3] Chen Y., Lei Y., Li Y., Yu Y., Cai J., Chiu M.H., Rao R., Gu Y., Wang C., Choi W. (2020). Strain engineering and epitaxial stabilization of halide perovskites. Nature.

[4] Li S., Larionov K.V., Popov Z.I., Watanabe T., Amemiya K., Entani S., Avramov P.V., Sakuraba Y., Naramoto H., Sorokin P.B. (2020). Graphene/Half-Metallic Heusler Alloy: A Novel Heterostructure toward High-Performance Graphene Spintronic Devices. Adv. Mater..

[5] Chappert C., Fert A., Van Dau F.N. (2007). The emergence of spin electronics in data storage. Nat. Mater..

[6] Burschka J., Pellet N., Moon S.J., Humphry-Baker R., Gao P., Nazeeruddin M.K., Grätzel M. (2013). Sequential deposition as a route to high-performance perovskite-sensitized solar cells. Nature.

[7] Sakoda M., Iida K., Naito M. (2018). Recent progress in thin-film growth of Fe-based superconductors: Superior superconductivity achieved by thin films. Supercond. Sci. Technol..

[8] Zeng S., Li C., Chow L.E., Cao Y., Zhang Z., Tang C.S., Yin X., Lim Z.S., Hu J., Yang P. (2022). Superconductivity in infinite-layer nickelate La1- xCaxNiO2 thin films. Sci. Adv..

[9] Zhang R.X., Sarma S.D. (2021). Intrinsic time-reversal-invariant topological superconductivity in thin films of iron-based superconductors. Phys. Rev. Lett..

[10] Engelmann J., Grinenko V., Chekhonin P., Skrotzki W., Efremov D., Oswald S., Iida K., Hühne R., Hänisch J., Hoffmann M. (2013). Strain induced superconductivity in the parent compound BaFe_2_As_2_. Nat. Commun..

[11] Kang J.H., Ryan P.J., Kim J.W., Schad J., Podkaminer J.P., Campbell N., Suttle J., Kim T.H., Luo L., Cheng D. (2021). Local atomic configuration control of superconductivity in the undoped pnictide parent compound BaFe_2_As_2_. ACS Appl. Electron. Mater..

[12] Tamir I., Benyamini A., Telford E., Gorniaczyk F., Doron A., Levinson T., Wang D., Gay F., Sacépé B., Hone J. (2019). Sensitivity of the superconducting state in thin films. Sci. Adv..

[13] Thomsen C., Strait J., Vardeny Z., Maris H.J., Tauc J., Hauser J.J. (1984). Coherent Phonon Generation and Detection by Picosecond Light Pulses. Phys. Rev. Lett..

[14] Vaswani C., Wang L.L., Mudiyanselage D.H., Li Q., Lozano P.M., Gu G.D., Cheng D., Song B., Luo L., Kim R.H. (2020). Light-Driven Raman Coherence as a Nonthermal Route to Ultrafast Topology Switching in a Dirac Semimetal. Phys. Rev. X.

[15] Wu S., Geiser P., Jun J., Karpinski J., Sobolewski R. (2007). Femtosecond optical generation and detection of coherent acoustic phonons in GaN single crystals. Phys. Rev. B.

[16] Pezeril T., Ruello P., Gougeon S., Chigarev N., Mounier D., Breteau J.M., Picart P., Gusev V. (2007). Generation and detection of plane coherent shear picosecond acoustic pulses by lasers: Experiment and theory. Phys. Rev. B.

[17] Huang Y.K., Chern G.W., Sun C.K., Smorchkova Y., Keller S., Mishra U., DenBaars S.P. (2001). Generation of coherent acoustic phonons in strained GaN thin films. Appl. Phys. Lett..

[18] Hurley D.H., Wright O.B., Matsuda O., Gusev V.E., Kolosov O.V. (2000). Laser picosecond acoustics in isotropic and anisotropic materials. Ultrasonics.

[19] Harb M., Peng W., Sciaini G., Hebeisen C.T., Ernstorfer R., Eriksson M.A., Lagally M.G., Kruglik S.G., Miller R.J. (2009). Excitation of longitudinal and transverse coherent acoustic phonons in nanometer free-standing films of (001) Si. Phys. Rev. B.

[20] Song B.Q., Yang X., Sundahl C., Kang J.H., Mootz M., Yao Y., Perakis I.E., Luo L., Eom C.B., Wang J. (2023). Ultrafast Martensitic Phase Transition Driven by Intense Terahertz Pulses. Ultrafast Sci..

[21] Kumar S., Harnagea L., Wurmehl S., Buchner B., Sood A.K. (2012). Acoustic and optical phonon dynamics from femtosecond time-resolved optical spectroscopy of the superconducting iron pnictide Ca(Fe_0.944_Co_0.056_)_2_As_2_. Europhys. Lett..

[22] Wang J., Hashimoto Y., Kono J., Oiwa A., Munekata H., Sanders G.D., Stanton C.J. (2005). Propagating coherent acoustic phonon wave packets in In_*x*_Mn_1−*x*_As/GaSb. Phys. Rev. B.

[23] Ruello P., Pezeril T., Avanesyan S., Vaudel G., Gusev V., Infante I.C., Dkhil B. (2012). Photoexcitation of gigahertz longitudinal and shear acoustic waves in BiFeO_3_ multiferroic single crystal. Appl. Phys. Lett..

[24] Ogi H., Fujii M., Nakamura N., Yasui T., Hirao M. (2007). Stiffened ultrathin Pt films confirmed by acoustic-phonon resonances. Phys. Rev. Lett..

[25] Matsuda O., Wright O.B., Hurley D.H., Gusev V.E., Shimizu K. (2004). Coherent shear phonon generation and detection with ultrashort optical pulses. Phys. Rev. Lett.

[26] Mela I., Poudel C., Anaya M., Delport G., Frohna K., Macpherson S., Doherty T.A.S., Scheeder A., Stranks S.D., Kaminski C.F. (2021). Revealing Nanomechanical Domains and Their Transient Behavior in Mixed-Halide Perovskite Films. Adv. Mater..

[27] Frazer T.D., Knobloch J.L., Hernández-Charpak J.N., Hoogeboom-Pot K.M., Nardi D., Yazdi S., Chao W., Anderson E.H., Tripp M.K., King S.W. (2020). Full characterization of ultrathin 5-nm low- k dielectric bilayers: Influence of dopants and surfaces on the mechanical properties. Phys. Rev. Mater..

[28] Hay J., Crawford B. (2011). Measuring substrate-independent modulus of thin films. J. Mater. Res..

[29] Nguyen H.K., Fujinami S., Nakajima K. (2016). Elastic modulus of ultrathin polymer films characterized by atomic force microscopy: The role of probe radius. Polymer.

[30] Song B.Q., Xu M., Borisov V., Palasyuk O., Wang C.Z., Valentí R., Canfield P.C., Ho K.M. (2021). Construction of heterolayer intermetallic crystals: Case studies of the 1144-phase TM-phosphides (TM) (TM = Fe, Ru, Co, Ni). Phys. Rev. Mater..

[31] Patz A., Li T., Ran S., Fernandes R.M., Schmalian J., Bud’Ko S.L., Canfield P.C., Perakis I.E., Wang J. (2014). Ultrafast observation of critical nematic fluctuations and giant magnetoelastic coupling in iron pnictides. Nat. Commun..

[32] Kimber S.A., Kreyssig A., Zhang Y.Z., Jeschke H.O., Valentí R., Yokaichiya F., Colombier E., Yan J., Hansen T.C., Chatterji T. (2009). Similarities between structural distortions under pressure and chemical doping in superconducting BaFe_2_As_2_. Nat. Mater..

[33] Song B.Q., Nguyen M.C., Wang C.Z., Canfield P.C., Ho K.M. (2018). Is it possible to stabilize the 1144-phase pnictides with tri-valence cations?. Phys. Rev. Mater..

[34] Iyo A., Kawashima K., Kinjo T., Nishio T., Ishida S., Fujihisa H., Gotoh Y., Kihou K., Eisaki H., Yoshida Y. (2016). New Structure Type Fe Based Superconductors: CaAFe_4_As_4_ (A = K, Rb, Cs) and SrAFe_4_As_4_ (A = Rb, Cs). J. Am. Chem. Soc..

[35] Johnston D.C. (2010). The puzzle of high temperature superconductivity in layered iron pnictides and chalcogenides. Adv. Phys..

[36] Zaikina J.V., Batuk M., Abakumov A.M., Navrotsky A., Kauzlarich S.M. (2023). Facile Synthesis of Ba_1−*x*_K_*x*_Fe_2_As_2_ Superconductors via Hydride Route. Crystallography Open Database. http://www.crystallography.net/cod/4123005.html.

[37] Zaikina J.V., Batuk M., Abakumov A.M., Navrotsky A., Kauzlarich S.M. (2023). Facile Synthesis of Ba_1−*x*_K_*x*_Fe_2_As_2_ Superconductors via Hydride Route. Crystallography Open Database. http://www.crystallography.net/cod/4123009.html.

[38] Wyckoff R. (2023). Interscience Publishers, New York, New York Rocksalt Structure. Crystallography Open Database. http://www.crystallography.net/cod/9008667.html.

[39] Dressler L., Griebner U., Kittner R. (1987). Precision measurement of lattice parameters in LiF monocrystals. Crys. Res. Technol..

[40] Momma K., Izumi F. (2011). VESTA 3 for three-dimensional visualization of crystal, volumetric and morphology data. Appl. Crystallogr..

[41] Wallace M.K., Winey J.M., Gupta Y.M. (2021). Sound speed measurements in lithium fluoride single crystals shock compressed to 168 GPa along [100]. J. Appl. Phys..

[42] Briscoe C.V., Squire C.F. (1957). Elastic Constants of LiF from 4.2K to 300K by Ultrasonic Methods. Phys. Rev..

[43] Dressel M., Grüner G. (2002). Electrodynamics of Solids: Optical Properties of Electrons in Matter.

[44] Dai Y.M., Akrap A., Bud’ko S.L., Canfield P.C., Homes C.C. (2016). Optical properties of AFe_2_As_2_ (A = Ca, Sr, and Ba) single crystals. Phys. Rev. B.

[45] Poirier M., Bilodeau M., Lefebvre S., Karki A.B., Jin R. (2014). Ultrasonic and microwave investigation of the structural and magnetic transitions in CaFe_2_As_2_ and BaFe_2_As_2_ single crystals. Phys. Rev. B.

[46] Fujii C., Simayi S., Sakano K., Sasaki C., Nakamura M., Nakanishi Y., Kihou K., Nakajima M., Lee C.H., Iyo A. (2018). Anisotropic Grüneisen parameter and diverse order parameter fluctuations in iron-based superconductor Ba(Fe_1−x_Co_x_)_2_As_2_. J. Phys. Soc. Jpn..

[47] Perdew J.P., Burke K., Ernzerhof M. (1996). Generalized Gradient Approximation Made Simple. Phys. Rev. Lett..

[48] Craig R.R., Taleff E.M. (1996). Mechanics of Materials.

[49] Hoffmann R., Zheng C. (1985). Making and breaking bonds in the solid state: The ThCr_2_Si_2_ structure. J. Phys. Chem..

[50] Abou Saleh A., Rudenko A., Douillard L., Pigeon F., Garrelie F., Colombier J.P. (2019). Nanoscale imaging of ultrafast light coupling to self-organized nanostructures. ACS Photonics.

[51] Stinson H., Sternbach A., Najera O., Jing R., Mcleod A., Slusar T., Mueller A., Anderegg L., Kim H., Rozenberg M. (2018). Imaging the nanoscale phase separation in vanadium dioxide thin films at terahertz frequencies. Nat. Commun..

[52] Kamboj V.S., Singh A., Ferrus T., Beere H.E., Duffy L.B., Hesjedal T., Barnes C.H., Ritchie D.A. (2017). Probing the topological surface state in Bi2Se3 thin films using temperature-dependent terahertz spectroscopy. ACS Photonics.

[53] de la Torre A., Seyler K.L., Buchhold M., Baum Y., Zhang G., Laurita N.J., Harter J.W., Zhao L., Phinney I., Chen X. (2022). Decoupling of static and dynamic criticality in a driven Mott insulator. Commun. Phys..

[54] Ke L., Zhang L., Zhang N., Wu Q.Y.S., Leong H.S., Abdelaziem A., Mehta J.S., Liu Y.C. (2022). Corneal elastic property investigated by terahertz technology. Sci. Rep..

[55] Juvé V., Crut A., Maioli P., Pellarin M., Broyer M., Del Fatti N., Vallée F. (2010). Probing elasticity at the nanoscale: Terahertz acoustic vibration of small metal nanoparticles. Nano Lett..

[56] Du X., Li J., Niu G., Yuan J.H., Xue K.H., Xia M., Pan W., Yang X., Zhu B., Tang J. (2021). Lead halide perovskite for efficient optoacoustic conversion and application toward high-resolution ultrasound imaging. Nat. Commun..

[57] Hou Y., Nichele F., Chi H., Lodesani A., Wu Y., Ritter M.F., Haxell D.Z., Davydova M., Ilić S., Glezakou-Elbert O. (2023). Ubiquitous superconducting diode effect in superconductor thin films. Phys. Rev. Lett..

[58] Luo L., Mootz M., Kang J.H., Huang C., Eom K., Lee J.W., Vaswani C., Collantes Y.G., Hellstrom E.E., Perakis I.E. (2023). Quantum coherence tomography of light-controlled superconductivity. Nat. Phys..

[59] Chen S., Yuan S., Hou Z., Tang Y., Zhang J., Wang T., Li K., Zhao W., Liu X., Chen L. (2021). Recent progress on topological structures in ferroic thin films and heterostructures. Adv. Mater..

[60] Thakur B., Kumar Y., Gupta M., Deshpande U., Shekar N.C., Chakravarty S. (2022). Investigating the effect of thickness on the structural and magnetic properties of carbon thin film. Carbon.

[61] Kant N., Singh P. (2022). Review of next generation photovoltaic solar cell technology and comparative materialistic development. Mater. Today Proc..

[62] Moon M.M.A., Ali M.H., Rahman M.F., Kuddus A., Hossain J., Ismail A.B.M. (2020). Investigation of thin-film p-BaSi_2_/n-CdS heterostructure towards semiconducting silicide based high efficiency solar cell. Phys. Scr..

[63] Li W., Shi J., Zhang K.H., MacManus-Driscoll J.L. (2020). Defects in complex oxide thin films for electronics and energy applications: Challenges and opportunities. Mater. Horiz..

[64] Ulyanenkov A. (2004). Leptos: A universal software for x-ray reflectivity and diffraction. Advances in Computational Methods for X-ray and Neutron Optics.

