# Supplementary material for: Study of Elastic and Structural Properties of BaFe2As2 Ultrathin Film Using Picosecond Ultrasonics
Source: Materials (Basel). 2023 Nov 3;16(21):7031. doi: 10.3390/ma16217031 (PMC10650054; doi:10.3390/ma16217031)
Supplement: Supplementary file 1 [file materials-16-07031-s001.zip › materials-2660573-supplementary.pdf]

Supplementary Materials: Study of elastic and structural properties in $BaFe_2As_2$ ultrathin film using picosecond ultrasonics

Di Cheng[1,2], Boqun Song[1,2], Jong-Hoon Kang[3], Chris Sundahl[3], Anthony L. Edgeton[3], Liang Luo[1,2], Joong-Mok Park[1,2], Yesusa G. Collantes[4], Eric E. Hellstrom[4], Martin Mootz[5], Ilias E. Perakis[5], Chang-Beom Eom[3], and Jigang Wang[1,2,*]

1 Department of Physics and Astronomy, Iowa State University, Ames, IA 50011, USA; dcheng@iastate.edu (D.C.); bqsong@iastate.edu (B.S.); liangluo@ameslab.gov (L.L.); joongmok@iastate.edu (J.-M.P.)
2 Ames National Laboratory-USDOE, Ames, IA 50011, USA
3 Department of Materials Science and Engineering, University of Wisconsin-Madison, Madison,WI 53706, USA; jkang@postech.ac.kr (J.-H.K.); sundahlc@gmail.com (C.S.); edgeton@wisc.edu (A.L.E.);
ceom@wisc.edu (C.-B.E.)
4 Applied Superconductivity Center, National High Magnetic Field Laboratory, Florida State University, Tallahassee, FL 32310, USA; hellstrom@asc.magnet.fsu.edu (E.E.H.)
5 Department of Physics, University of Alabama at Birmingham, Birmingham, AL 35294-1170, USA;
mootz@iastate.edu (M.M.); iperakis@uab.edu (I.E.P.)
* Correspondence: jgwang@iastate.edu or jgwang@ameslab.gov

We present the details of thickness determinations for nominal 9 nm and 60 nm $BaFe_2As_2$ (Ba-122) thin films grown by pulsed laser deposition (PLD) technique. The thickness of the 9 nm Ba-122 thin film is precisely determined by fitting its X-ray reflectivity (XRR) spectrum using LEPTOS[1] software. The simulated sample modeled is a Ba-122 thin film on a LiF substrate with density, roughness, and Ba-122 thickness left as free variables. The genetic algorithm was used as an optimization algorithm to find the global minimum. As is shown in Figure S1(a), the fitting range for the 9 nm film is restricted to 2θ angles below which clear Kiessig fringes persist. We obtained 9.1 ± 0.1 nm based on the fitting (other fitted parameters: density 9.45 ± 0.30 g/cm^3, roughness 0.25 ± 4.00 nm). Error reported is the standard deviation determined from the variance in the fitting parameters from best-fit calculations.

As for the 60 nm nominal Ba-122 thin film, no observable Kiessig fringes were observed in Figure S1(a). To determine the thickness we performed transmission electron microscopy (TEM) measurement on it to determine its thickness (Figure S1(b)). the thickness of the 60 nm nominal Ba-122 film, based on Figure S1(b), is 62.4 ± 1.2 nm. The error reported is the standard deviation determined from the 20 equally spaced regions (shown in Figure S1(b) inset).

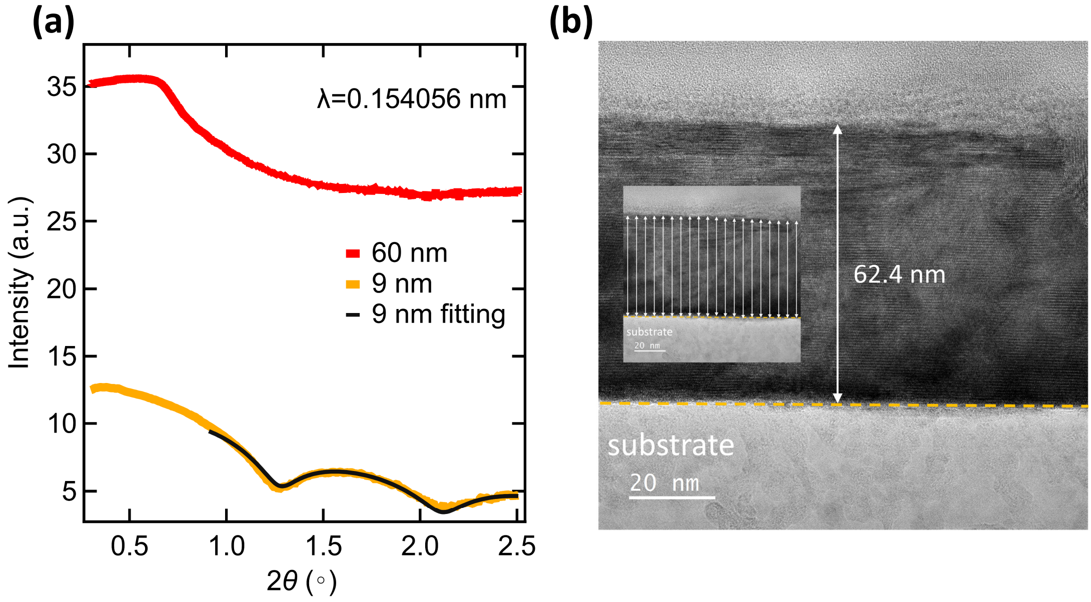

Figure S1. Thickness determinations of Ba-122 thin films. (**a**) X-ray reflectivity spectra and corresponding fittings for nominal 9 nm and 60 nm Ba-122 thin films. (**b**) TEM image of 60 nm Ba-122 thin film. Inset shows the equally spaced positions for deviation determination.

References

[1] A. Ulyanenkov, “Leptos: a universal software for x-ray reflectivity and diffraction,” *Advances in Computational Methods for X-Ray and Neutron Optics*, vol. 5536, pp. 1–15, 2004.
